# Subarachnoid Hemorrhage on Abdominal Computed Tomography

**DOI:** 10.5334/jbsr.1680

**Published:** 2019-01-30

**Authors:** Yukari Wakabayashi, Goshi Takahashi

**Affiliations:** 1 Kohseichuo General Hospital, JP; 2 Toho University Ohashi Medical center, JP

**Keywords:** Subarachnoid hemorrhage, Abdominal computed tomography, Spinal canal

## Report

A 54-year-old woman visited our emergency department because of repeated vomiting for one day. Physical examination and blood analysis revealed subtle tenderness of the right upper abdomen and mild elevation of white blood cell count and C-reactive protein level. She underwent pre- and post-contrast abdomino-pelvic computed tomography (CT). A small gallstone was detected, and slight edema of the gallbladder was suspected. She was admitted with the suspicion of mild cholecystitis.

**Figure 1:**
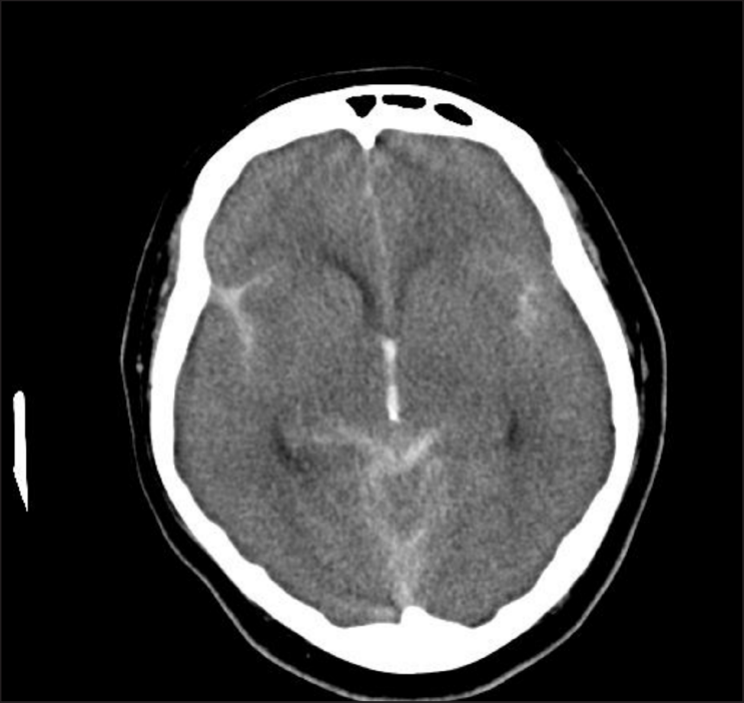


**Figure 2:**
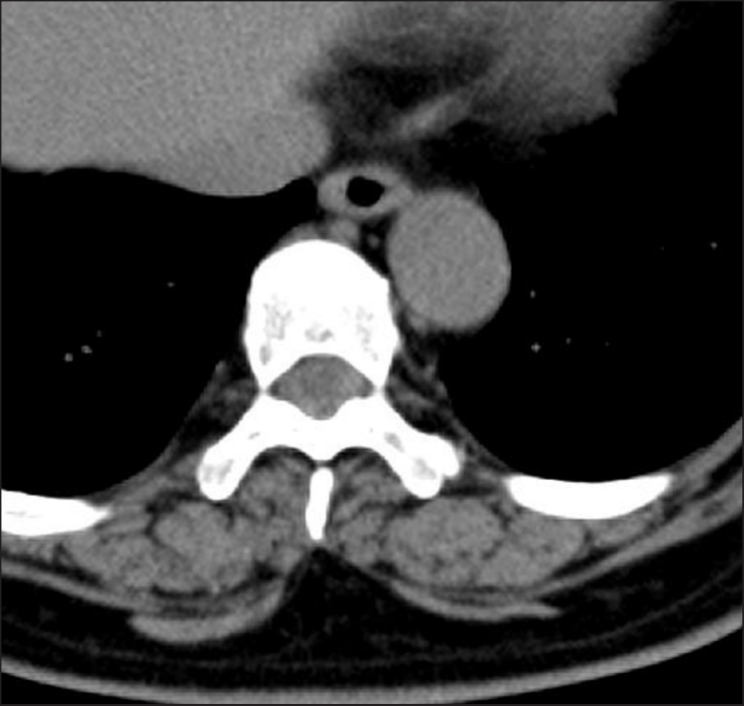


On the morning after the admission, she reported headache and collapsed. Brain CT revealed a large subarachnoid hematoma with hydrocephalus (Figure 1). Considering the distribution of the hematoma and the existence of hydrocephalus, repeated aneurysm rupture was suspected. The abdomino-pelvic CT data were re-assessed, and increased density of the cerebrospinal fluid (CSF) (more than that of the spinal cord) was noted in the spinal canal (Figure 2). We suspected that the first rupture occurred before the admission. Her family confirmed that she experienced relatively severe headache three days prior to admission.

## Comment

The symptoms of non-traumatic subarachnoid hemorrhage (SAH) are usually sudden and include severe headache. If brain CT is performed within 24 hours of onset, SAH can be diagnosed in over 90% of cases. However, with time, SAH becomes more difficult to detect on CT because of CSF clearance [1]. When the initial rupture is not severe and the headache is mild, the onset is usually overlooked, and the patient visits the hospital for other vague symptoms or visits the emergency room only after a second rupture. In this setting, if previous body CT data are available that show SAH within the spinal canal, the findings may help speculating the onset of aneurysm rupture.

Conversely, if SAH is incidentally found within the spinal canal, the most frequent cause is traumatic medical procedures, such as lumbar puncture and surgery, and it is followed by intracranial SAH. In patients without a history of recent lumbar puncture or surgery, SAH in the spinal canal may be the first finding of intracranial SAH.
